# Acute flares of knee osteoarthritis in primary care: a feasibility and pilot case-crossover study

**DOI:** 10.1186/s40814-018-0359-4

**Published:** 2018-11-05

**Authors:** Martin J. Thomas, Stephanie Butler-Walley, Trishna Rathod-Mistry, Zoe Mayson, Emma L. Parry, Christopher Pope, Tuhina Neogi, George Peat

**Affiliations:** 1 Arthritis Research UK Primary Care Centre, Research Institute for Primary Care & Health Sciences, Keele University, Keele, Staffordshire ST5 5BG UK; 2 Haywood Academic Rheumatology Centre, Midlands Partnership NHS Foundation Trust, Haywood Hospital, Burslem, Staffordshire ST6 7AG UK; 3 Keele Clinical Trials Unit, David Weatherall Building, Keele University, Keele, Staffordshire ST5 5BG UK; 4 Department of Medicine, Sections of Clinical Epidemiology Research and Training Unit, and Rheumatology, Boston University School of Medicine, 650 Albany Street, Clinical Epidemiology Unit, Suite X-200, Boston, MA 022118 USA

**Keywords:** Knee, Osteoarthritis, Flare, Web-based, Case-crossover

## Abstract

**Background:**

Osteoarthritis (OA) is a leading cause of persistent pain and disability. Traditionally viewed as a slowly progressive disease, the impact of symptom variability on prognosis remains unclear. ‘Acute-on-chronic’ episodes are a well-recognised feature of many long-term conditions but only recently formally described in OA. This study aimed to develop a web-based data collection platform and establish key methodological design parameters, to develop a larger community-based study investigating acute flares of knee OA in England.

**Methods:**

The study is a 9-week feasibility and pilot web-based observational case-crossover study. Adults aged ≥ 40 years registered with two general practices who had consulted their general practitioner for knee pain/OA in the last 2 years were recruited. Participants completed a baseline questionnaire and scheduled (control-period) questionnaires at follow-up weeks 1, 5, and 9. Participants were invited to self-declare via the website on any occasion they experienced a knee pain flare-up lasting ≥ 24 h. Upon notification, an event-driven (case-period) questionnaire comparable to the scheduled questionnaires was completed and daily measurements on the course and consequences were taken until resolution. A sub-study of 10 participants logged daily pain measurements. The analysis estimated key parameters including recruitment (selective non-participation, eligibility, consent), retention, and flare-up capture processes. Questionnaire completeness and website usability were evaluated.

**Results:**

Of 442 patients invited, 14 completed baseline questionnaires. Eligibility rate was 26.9% (95% CI 19.3, 36.2), consent rate 53.6% (35.8, 70.5), and overall recruitment rate 3.2% (1.9, 5.2). Compared to those mailed, baseline responders were more likely to be male and ≥ 65 years, as were those reporting ≥ 1 flare-up. Eleven scheduled questionnaires were completed (mean response 35%). Although seven participants (50%) self-declared 11 flare-ups, only one event-driven questionnaire was completed and three participants contributed daily flare measurement for four flares. Missing data was ≤ 3.7% across completed baseline, scheduled, and event-driven questionnaires. Aspects of website usability require minor refinement.

**Conclusions:**

Recruitment was not feasible with the current strategy. An evaluation of processes has suggested several substantial changes in design that may enhance recruitment, retention, and data quality in a future full-scale study.

## Background

Osteoarthritis (OA) is a leading cause of disability in populations worldwide [1]. In the UK each year, an estimated 4% of adults aged ≥ 45 years consult their general practice with a recorded diagnosis of OA [2]—equating to more than a million primary care consultations each year [3].

Osteoarthritis has typically been characterised as a progressive, non-inflammatory disease emerging from middle age onwards in response to exposures earlier in life (e.g. severe injury, cumulative excess mechanical loading), and whose course is marked by slow, steady decline. Despite this, there is increasing recognition that ‘acute-on-chronic’ episodes and ‘flare-ups’ of more severe pain are part of the natural history [4–6], although fundamental questions remain unanswered about these phenomena.

Understanding flare-ups in OA is important for several reasons: they can be distressing and disabling [4] and they may drive patterns of intermittent healthcare use, including over-the-counter analgesic and non-steroidal anti-inflammatory use, primary care consultation, and intra-articular injection. Dramatic changes in symptom severity appear to disrupt healthy behaviours such as maintaining a healthy weight and a physically active lifestyle, which are regarded as important for the long-term management of OA [7]. In other long-term conditions, where acute exacerbations are a recognised feature of the natural history [8–12], research has provided important insights into groups to target (e.g. ‘frequent exacerbator phenotype’ in Chronic Obstructive Pulmonary Disease (COPD) [13–15]), as well as underpinning the discovery and evaluation of effective biomedical [16] and behaviour change, and service organisation [17] interventions.

Within a programme of research into flare-ups in OA, we sought to design and undertake a full-scale web-based case-crossover study to estimate proximate triggers of acute flares in knee OA, determine their course and consequences, and identify high-risk patient profiles that will direct future prevention and management for patients and healthcare practitioners. Case-crossover studies [18] have been used to good effect to identify triggers of other acute health events (e.g. [19–21]), and researchers have begun adopting this design to investigate episodic flare-ups of OA [22]. However, there are a number of challenges and uncertainties, including the ability to identify and recruit individuals at risk of flare-ups to online data collection, efficient and timely capture of these relatively short-lived and currently ill-defined events, and the ascertainment of relevant, transient exposures. We undertook a feasibility and pilot web-based observational case-crossover study with the purpose of informing a future full-scale case-crossover study.

### Overall aim

The overall aim of this feasibility and pilot study was to establish important parameters and test several processes that would inform the design of a future web-based observational case-crossover study of acute flares in knee OA.

### Objectives

Specific objectives of the study were to:

i. Establish the feasibility of the recruitment strategy (including willingness of patients to make the transition from offline to online research participation and evidence of selective non-participation and retention)
ii. Clarify the suitability of the study eligibility criteria
iii. Establish the completeness of the data ascertained
iv. Estimate the proportion of participants reporting a flare-up during a 9-week observation period
v. Explore the feasibility of processes for nesting methodological sub-studies in a future full-scale study, including the potential willingness of participants to provide biomarker data
vi. Identify any improvements needed for functionality and usability of the study website and email support

## Methods

### Study design and setting

This feasibility and pilot study is a community web-based observational case-crossover study [18], a design which focusses on within-person comparisons (‘Why now?’ rather than ‘Why me?’ [23]), by comparing the relative frequency of brief intermittent exposures (potential triggers) in periods just before transient acute health events or episodes of interest [24, 25]. Applied in the present context, the design seeks to estimate the direction and magnitude of associations between a range of short-lived exposures and flare-ups of knee OA. By conducting the study online, we hoped to have a relatively low-cost design, capable of obtaining retrospective exposure data as close as possible to the onset of a flare-up, and of sampling (control-period) exposure frequency among participants by repeated scheduled measurements. Ethical approval for the study was obtained from North East – York Research Ethics Committee (REC Reference number: 16/NE/0390).

### Participant identification and recruitment

Potentially eligible adults with knee OA were identified and recruited from two local general practices in North Staffordshire, England. Table 1 summarises the participant inclusion and exclusion criteria.

**Table 1:** Eligibility criteria

Inclusion criteria	Mode of ascertainment
Male or female aged ≥ 40 years	GPSS
Registered as a permanent resident with participating general practices	GPSS
Recorded consultation for knee OA or knee OA-related joint symptoms in the last 2 years^†^	GPSS
Access to an email account and to the Internet	PCRF
Exclusion criteria	
Known diagnosis of inflammatory arthropathy, spondyloarthropathy, or crystal arthropathy (e.g. rheumatoid arthritis, ankylosing spondylitis, reactive arthritis, systemic lupus erythematosus, gout, psoriatic arthritis)†	GPSS
Recorded diagnosis of fibromyalgia†	GPSS
Red flags: recent significant trauma to knees; acutely hot swollen joint	GPSS, PCRF
Previous total knee arthroplasty or on waiting list for total knee arthroplasty	GPSS, PCRF
Corticosteroid injection into the knee in the last 3 months	PCRF
Surgery to either knee within the past 3 months	GPSS, PCRF
Unable to complete questionnaires written in English via the study website	PCRF
Vulnerable individuals (e.g. psychiatric illness, learning difficulties, dementia, terminal illness, and severe enduring mental ill health)	GPSS

*GPSS* general practice search and screen, *PCRF* patient-completed reply form

^†^Based on code lists (available at http://www.keele.ac.uk/mrr/morbiditydefinitions/)

All eligible participants were mailed a study pack (invitation letter on General Practice headed paper, participant information sheet (PIS), reply form with eligibility questions and request for contact email address, and pre-paid return envelope). Non-responders to the initial invitation were sent a reminder 2 weeks later. Individuals who returned a completed reply form, fulfilled the eligibility criteria, and provided a valid personal email address were sent a welcome email containing a link to the study website, which was hosted on a secure network. Eligible participants accessing the link were asked to confirm they had read and understood the PIS and were then invited to provide informed electronic consent (e-Consent) to take part in the study. Consenting participants were directed to a login page to set up their unique username and password.

### Data collection

Data collection comprised four main strands: a baseline questionnaire to collect descriptive characteristics from participants, scheduled questionnaires to collect information on exposure frequency on days not followed by a flare-up, event-driven questionnaires to collect information on flare-ups and exposure frequency on the days prior to a flare-up, and ultra-short daily flare-up questionnaires to be completed following flare notification until its resolution. Once any questionnaire was completed and submitted, participants had no repeat access to their answers. All questionnaires had to be completed at one time point, and there was no facility for partial completion and return at a later time.

#### Baseline questionnaire

Upon activating a website login, participants were directed to the baseline questionnaire (although they could opt to complete this later via an emailed web link) and sent a welcome email including information about logging in and how to self-report a flare-up (see below). Email reminders were sent to participants who had not completed their baseline questionnaire after 3 days and again after a further 3 days. On day 8, the questionnaire became deactivated and an email was sent notifying participants that they could not continue in the study. Table 2 presents the baseline questionnaire content.

**Table 2:** Study questionnaires

Concept	Measurement method	Detail	Time available for completion	Reminder sent
Baseline questionnaire				
Section A: knee pain	Duration	Pain in last 12 months. Left, right	7 days	Yes
	Time since onset	< 1 year, 1–4 years, 5–9 years, 10+ years. Left, right		
	Pattern [36]	5 flare pattern illustrations. Left, right		
	Experience of knee pain [37]	In past 6 months: no pain, predictable pain, some unpredictability, constant. Left, right		
	Walking difficulty [38]	Injury induced walking problems for at least 1 week. Left, right		
	Pain, aching, stiffness in last month [39, 40]	No days, few days, some days, all days. Left, right		
	Worst/least in last week, average, current [41]	0–10 NRS with anchors (no pain, pain as bad as you can imagine)		
	Flare-up at present	Yes/no. Left, right		
	Self-reported main flare trigger	Free text		
	Bothersomeness in last 24 h [42]	Not at all, slightly, moderately, very much, extremely. Left, right		
	Leg angles [43]	Very bow legged, bowed legged, normal, knock-knee, very knock-knee. Left, right		
	Foot angles [43]	Very turned out feet, turned out feet, straight, turned in feet, very turned in feet. Left, right		
	KOOS-PS [44]	7-item and 5-option categories for difficulties with daily activities in last week		
	KOOS-Qol [45]	4-item and 5-option categories for quality of life in last week		
	Medications for knee pain, last week Health professional consultation for knee pain, last year	17-option categories for drug use General practitioner, practice/district nurse, physiotherapist, surgeon, rheumatologist, acupuncturist, occupational therapist.		
Section B: general health	Perceived general health [46] Physical activity(GPPAQ) [47]	Excellent, very good, good, fair, poor Work physical activity (5-response options), general physical activity in last week (5-response options, 4-option categories), walking pace		
	Self-reported weight	Stones/lbs. or kg		
	Self-reported height	Feet/inches or cm		
Section C: demographics	Gender Date of birth Current employment	Male/female Date/month/year Paid employment or self-employed, retired, looking after home and/or family, unable to work because of sickness or disability, unemployed, doing unpaid or voluntary work, full or part-time student		
Scheduled (control-period) and event-driven (case-period) questionnaires*				
Knee pain	Flare-up at present	Yes/no. Left, right	7 days/ 2 days	Yes
	Average pain in last 24 h [27]	0–10 NRS with anchors (no pain, pain as bad as you can imagine). Left, right		
Changes noticed since flare-up†	Limping, swelling, stiffness, increased difficulty with activities of daily living, sleep disturbed by knee pain	Tick as many boxes as apply		
Physical activities	Vigorous physical activity > 10 mins [48]	Yes/no		
	Climbing several flights of stairs	Yes/no		
	Repetitive or prolonged squatting	Yes/no		
	Repetitive or prolonged kneeling	Yes/no		
	Climbing up and down ladders	Yes/no		
	Lifting or moving heavy objects	Yes/no		
	Prolonged periods of sitting without a break	Yes/no		
	Prolonged periods of standing without a break	Yes/no		
	Any unusual activities involving knees	Yes/no		
Slips, trips, sprains, strains	Slip, trip, or fall Episode of buckling or giving way [49]	Yes/no Yes/no. Left, right		
Health and healthcare	Reduce or miss medication Take extra medication	Yes/no Yes/no		
	Cough, cold, other minor infection	Yes/no		
Stress and other things	Work related stress [50] Home related stress [50]	Yes/no Yes/no		
	Friend/family-related stress [50]	Yes/no		
	Low mood/depression	Yes/no		
	Feeling angry, irritable, hostile	Yes/no		
	Poor night’s sleep	Yes/no		
	Eat foods usually avoided	Yes/no		
	Cold/damp weather	Not at all, slightly, moderately, severely, extremely		
Day of above exposure	All yes responses from above sections (physical activity; slips, trips, sprains, strains; health and healthcare; stress and other things) anchored to day of the week	Grid of previous 7 days from declared flare-up onset. Tick as many boxes as apply		
Brief daily questionnaire during flare-up				
Knee pain	Average pain in last 24 h [27]	0–10 NRS with anchors (no pain, pain as bad as you can imagine). Left, right	6 h	No
Impact of pain	Bothersomeness in last 24 h [42]	Not at all, slightly, moderately, very much, extremely. Left, right		
Medication use	Pain medication taken in last 24 h	No; yes, but less than usual; yes, but about the same as usual; yes, more than usual		
Flare-up resolution	Has your flare-up ended	Yes/no		
Brief daily questionnaire for sub-study				
Main flare-up trigger	Did your main trigger for a flare-up of knee pain [baseline response reminder] happen today	Yes/no	6 h	No
Knee pain	Average pain in last 24 h [27]	0–10 NRS with anchors (no pain, pain as bad as you can imagine). Left, right		

*KOOS-PS* Knee injury and Osteoarthritis Outcome Score-Physical Function Short Form, *Qol* qualify of life, *GPPAQ* General Practice Physical Activity Questionnaire, *NRS* numerical rating scale

*Control-period questionnaires scheduled for week 1, 5, and 9 post-baseline completion; event-driven questionnaires initiated by participant if and when flare-ups occur

^†^Question applied to event-driven questionnaires only

#### Scheduled (control-period) questionnaires

A scheduled control-period questionnaire (Table 2) asking questions about selected exposures during the last 7 days was sent to participants via an emailed web link 1 week, 5 weeks, and 9 weeks after completion of the baseline questionnaire. At the same time each questionnaire also became accessible on the study website, should participants login to the website independently. For scheduled control-period questionnaires, we followed the same process of email reminders and deactivating the questionnaire as per baseline questionnaire except that after deactivation, participants continued through the study to the next scheduled follow-up questionnaire.

The first question of the scheduled control-period questionnaire asked participants if they felt they were currently experiencing a flare-up. If they responded ‘yes’ to this question, they were immediately redirected to complete an event-driven questionnaire (see the ‘Event-driven (case-period) questionnaires’ section below). If participants indicated they were exposed to any of the selected exposures, they were taken to a calendar template to determine the date of exposure. For this template, the last 7 days were calculated from the present date, and participants were invited to select which days over this period they were exposed to each of their selected exposures.

#### Event-driven (case-period) questionnaires

Participants were invited to complete an event-driven flare-up case-period questionnaire immediately, at any point if they provided notification through the study website that they were currently experiencing a flare-up. This was either via a web link provided in previous email correspondence (welcome and scheduled questionnaire emails) or by logging onto the study website and clicking a prominent flare notification icon. Participants were given the option to complete the questionnaire immediately or later by requesting an emailed web link to the questionnaire. The first question of the event-driven questionnaire asked participants to enter the date the flare-up started. If it was ‘today’, an onscreen notification was generated thanking participants before inviting them to notify us again tomorrow should their flare-up persist for 24 h. If it was 4 or more days previously, an onscreen notification was generated thanking the participant for their notification and informing them that there was no requirement for an event-driven questionnaire to be completed at this time. These participants were still invited to complete a short daily questionnaire via email until the resolution of their flare-up episode (see the ‘Daily flare-up questionnaires’ section below). If the flare-up started within the last 3 days, questions about activities during the last 7 days were taken from the date of onset. The system included the appropriate days of the week for each of the last 7 days of questioning to aid recall. If participants did not respond, an email reminder was sent after 1 day. If non-response continued, a repeat email reminder was sent after a further 1 day. If no response was received after 2 days, on day 3, the questionnaire became deactivated. Participants were notified of this by email. If participants did not complete an event-driven questionnaire, they continued through the study to the next scheduled follow-up questionnaire.

The content of the event-driven questionnaires was identical to the scheduled control-period questionnaire with the following exceptions: there was no question on flare-up at present, and there were questions on the knee affected by a current flare. These included changes noticed since the flare-up—limping, swelling, stiffness, increase difficulty with activities of daily living, and sleep disturbed by knee pain (Table 2).

#### Brief daily questionnaires during flare-up

Following completion of an event-driven questionnaire, the next day, participants were invited by email to answer brief daily questions about the flare-up (Table 2) until participants’ self-reported that their symptoms had returned to their pre-flare ‘normal’ state for 2 consecutive days. There were no daily reminders, and questions could only be completed on the day of invitation, with any earlier incomplete dates being deactivated. Emails were sent at 18:00 Greenwich Mean Time (GMT) and remained open until 23.59 each day for the duration of the flare-up episode. End-of-day reporting of pain has previously been shown to adequately represent average pain levels across the same day [26]. Participants still experiencing a flare-up at the end of the study period were not followed up beyond this time point.

#### Nested methodological sub-study questions

A nested sub-study involved the first 10 enrolled participants, who were invited to answer additional brief daily questions for the 9-week study period. These commenced following completion of the baseline questionnaire. The purpose of this sub-study was to obtain prospective data that could be examined relative to the retrospective data collected within the main scheduled and event-driven questionnaires. Questioning for each participant was individualised, based on their response in the baseline questionnaire about what they personally perceived to be their main trigger for a flare-up of knee pain. Brief questioning also asked about pain intensity in the last 24 h [27] (Table 2). There were no daily reminders, and questions could only be completed on the day of invitation, with any earlier incomplete dates being deactivated. If participants attempted to click on any out-of-date email links, they were automatically taken to the current day’s questionnaire, or directed to complete future questionnaires at the next appropriate time point. Emails were sent at 18:00 GMT and remained open until 23.59 each day for the duration of study.

Upon study completion, participants were asked whether, in principle, they would be willing to provide additional biomarker data through magnetic resonance imaging (MRI) and/or the provision of a synovial fluid sample via knee joint aspiration during a flare-up.

Objective weather data downloaded from the UK Meteorological Office for the study period supplemented subjective weather-based questioning.

### Patient involvement

Our study was a patient-confirmed research priority. A patient advisory group (PAG) assisted with the questionnaire content, website development, and post-study evaluation in the following ways: group workshops, written and verbal feedback of questionnaires and patient facing documentation, practical hands-on trials during website development, participation in website video clips of flare-up description, and interpretation of data. One member of the PAG also actively contributed as a member of the study management group (CP).

### Outcome definition

Our working definition of a self-reported flare-up of symptomatic knee OA was ‘an event in the natural course of the condition, characterised by a change in the patient’s baseline pain that is beyond normal day-to-day variation, sustained for at least 24 hours, and is sudden or quick in onset. It may impact on the ability to perform everyday activities and have resulted in an increase in analgesic intake’. The choice of a qualitative approach to definition, self-assessment, and the definition itself was informed by a previous systematic literature review [unpublished data at time of submission], group discussions with patients and members of the public, and findings from an earlier survey and 3-month pen-and-paper daily diary study [unpublished data at time of submission].

### Sample size

Based on our recently completed pen-and-paper daily diary study in a similar patient population and sample frame, and an assumed combined response and consent rate of 12%, we estimated 400–450 eligible participants would need to be screened from up to four average-sized general practices to provide the target sample of 50 participants deemed sufficient for the study objectives.

### Statistical analysis

As a feasibility and pilot study, the statistical analysis plan focussed on process measures and evaluative methods to inform a future full-scale study. A flowchart summarised the recruitment and retention of participants into the study, and descriptive statistics were used to estimate recruitment, eligibility, consent, and retention rates and to examine the extent of selective non-participation and the quality and completeness of the data ascertained across all study time points.

To examine and quantify the effect of self-reported recall bias of exposure triggers, comparative objective data collection was planned for one variable. Weather-based questioning asked at each scheduled and event-driven data collection time point could be compared with objective weather data downloaded from the UK Meteorological Office for the same period. This step can explore the feasibility of providing additional quantitative estimates adjusting for any inherent self-reporting recall bias.

All analyses were conducted using STATA V.14 (Stata Corporation, TX, USA).

## Results

### Study population

**Fig. 1:**
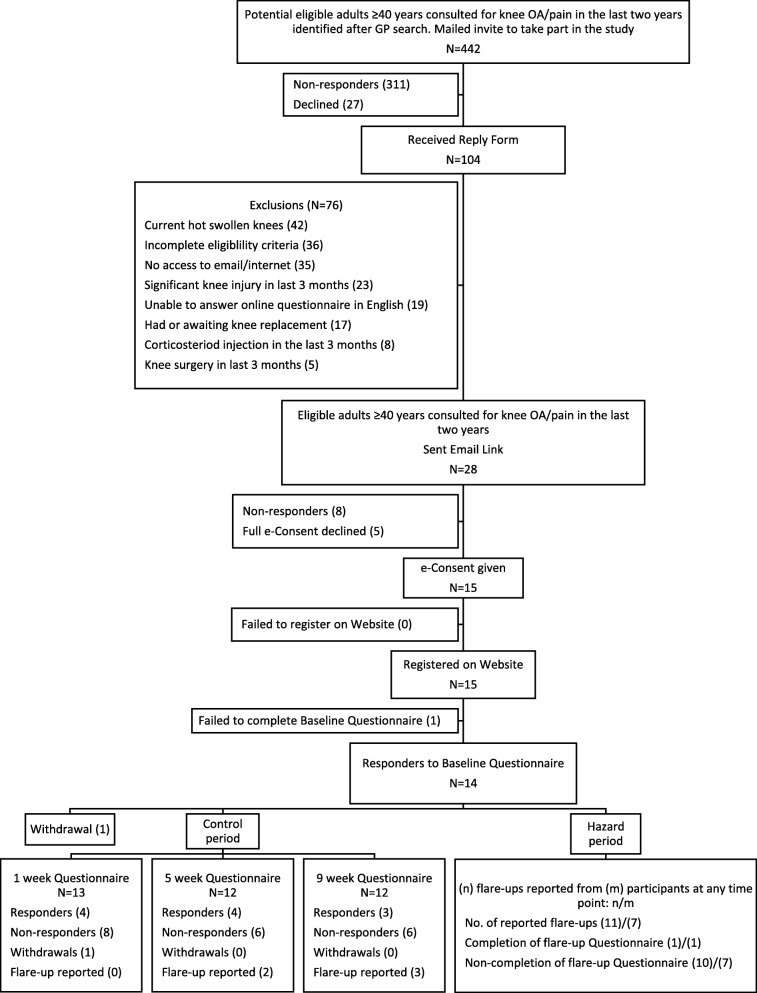
Flowchart of participants recruited into study

During March and April 2017, 442 adults aged 40 years and over were mailed a study invitation (Fig. 1). In total, 104 reply forms were received (crude response 23.5% (95% confidence intervals (CI) 19.8, 27.7)), of whom 28 were deemed eligible (eligibility rate among responders 26.9%; 19.3, 36.2) and 15 consented (consent rate among eligible responders 53.6%; 35.8, 70.5), all of whom registered an online account on the study website. There were 311 non-responders to invitation. In total, 14 people completed a baseline questionnaire, producing an overall recruitment rate of 3.2% (1.9, 5.2). Two participants withdrew from the study, one following completion of the baseline questionnaire and one following the week 1 scheduled questionnaire. Eight participants completed at least one scheduled follow-up. Using this as a follow-up indicator, estimated retention was 57.1% (32.6, 78.6).

### Selective non-participation

Compared with the initial mailed population, baseline responders appeared more likely to be male and aged ≥ 65 years (Table 3). There were insufficient numbers to meaningfully evaluate participant characteristics related to post-baseline attrition.

**Table 3:** Gender and age differences between exclusions, non-responders, and responders at each time point

	All mailed participants (*N* = 442)	Non-responders and refusals (*N* = 338)	Exclusions (*N* = 76)	Eligible (*N* = 28)	Eligible but dropped out (*N* = 14)	Baseline responders (*N* = 14)	Responders to at least one scheduled follow-up (*N* = 8)	Reported flares (*N* = 7)
Gender								
Males	199 (45)	152 (45)	30 (40)	17 (61)	8 (57)	9 (64)	6 (75)	5 (71)
Females	243 (55)	186 (55)	46 (61)	11 (39)	6 (43)	5 (36)	2 (25)	2 (29)
Age								
40–54	129 (29)	108 (32)	13 (17)	8 (29)	5 (36)	3 (21)	0 (0)	0 (0)
55–64	125 (28)	99 (29)	16 (21)	10 (36)	6 (43)	4 (29)	4 (50)	2 (29)
65+	188 (43)	131 (39)	47 (62)	10 (36)	3 (21)	7 (50)	4 (50)	5 (71)
Age, males								
40–54	64 (32)	52 (34)	6 (20)	6 (35)	4 (50)	2 (22)	0 (0)	0 (0)
55–64	61 (31)	47 (31)	7 (23)	7 (41)	4 (50)	3 (33)	3 (50)	2 (40)
65+	74 (37)	53 (35)	17 (57)	4 (24)	0 (0)	4 (44)	3 (50)	3 (60)
Age, females								
40–54	65 (27)	56 (30)	7 (15)	2 (18)	1 (17)	1 (20)	0 (0)	0 (0)
55–64	64 (26)	52 (28)	9 (20)	3 (27)	2 (33)	1 (20)	1 (50)	0 (0)
64+	114 (47)	78 (42)	30 (65)	6 (55)	3 (50)	3 (60)	1 (50)	2 (100)

Absolute numbers and percentages are provided

### Completeness and quality of data ascertained

Levels of missing item-level data were low among those participants who began each type of questionnaire. Of the 14 participants who began the baseline questionnaire, the median number of missing responses was 2 of 94 items (range 0–12). With the exception of one item for one participant, there were no missing item-level data from the 11 scheduled questionnaires completed by eight participants. The sole event-driven questionnaire that was completed had responses to only five of the physical activity exposure questions missing.

Eleven flare-up notifications were reported by seven participants during the study period (Table 4). Only one participant-reported flare-up met the study case definition and was therefore eligible to complete the flare-up questionnaire. For those who completed the daily flare-up questionnaire, two were monitored to resolution. These ended seven, and 23 days after the date, the flare-up was reported.

**Table 4:** Descriptive characteristics of flare-up capture and monitoring

Participant ID	Flare number	Flare captured by which questionnaire	Flare-up met study definition; reason if not		Flare ended	Completed flare-up questionnaire
XX526	1	Event-driven	No	Flare reported on day of onset	–	–
	2	Scheduled (week 9)	No	Flare reported on day of onset	–	–
XX581	1	Scheduled (week 5)	–	Day of onset missing	Missing	–
	2	Scheduled (week 9)	No	> 3 days since flare onset	Unresolved	–
XX637	1	Event-driven	No	> 3 days since flare onset	–	–
XX041	1	Event-driven	No	Flare reported on day of onset	–	–
XX065	1	Scheduled (week 5)	No	> 3 days since flare onset	23 days after date flare reported	–
	2	Scheduled (week 9)	No	> 3 days since flare onset	Unresolved	–
XX706	1	Event-driven	No	Flare reported on day of onset	–	–
XX160	1	Event-driven	No	Flare reported on day of onset	–	–
	2	Event-driven	Yes	–	7 days after flare reported	Yes

### Nested methodological sub-studies

For the first 10 participants recruited into the study, an exposure question was asked about their self-reported main trigger, together with daily measurements of knee pain for the 9-week study period. Four participants did not respond to any of the daily sub-study questionnaires, with the remaining six participants completing between 23 and 50 daily questionnaires from a possible 63 (Table 5). For two participants, there appeared little or no day-to-day variability in exposure to their main reported trigger during the study period.

**Table 5:** Daily pain measurements

Participant ID	Number of days questionnaire completed*	Number of days main trigger reported	Verbatim self-reported main trigger	Daily left knee pain severity Median (IQR)	Daily right knee pain severity Median (IQR)
XX526	27	11	Rising from sitting position	0 (0, 2)	0 (0, 2)
XX581	44	43	Stiff walking/pressure	1 (1, 1)	3 (2, 4)
XX592	49	6	Physical activity, cold damp weather	2 (1, 3)	2 (1, 3)
XX595	23	9	If I kneel down on it	0 (0, 0)	2 (0, 4)
XX599	0	–	‘Jarring’ of knee due to tiredness/stiffness following exercise	–	–
XX637	0	–	I do not know, but think it may be the weather	–	–
XX669	0	–	Cold damp weather, excess housework and gardening	–	–
XX701	0	–	Cannot pin point it	–	–
XX041	42	0	Over activity	1 (0, 1)	1 (0, 1)
XX065	50	41	I find that my knee pain can come at any time. I do the same things everyday but some days it is a bit more difficult to do as the pain gets in the way.	6 (5, 6)	6 (5, 7)

*IQR* interquartile range

*Participants were able to fill in the daily questionnaire for a maximum of 63 days

Six participants returned a post-study evaluation questionnaire. Four expressed willingness, in principle, to have an MRI scan during a flare-up, and two confirmed willingness to undergo a knee joint aspiration during a flare-up, as part of a future study.

There was insufficient response to both the scheduled and event-driven questionnaires to run an analysis to evaluate the comparison of subjective weather recall with objective UK Meteorological Office weather data.

## Discussion

This feasibility and pilot study has proved valuable for improving and refining processes and procedures for a future full-scale study. The study recruitment method, eligibility criteria, and processes to facilitate follow-up retention and enable flare notification, together with some key aspects of the web-based functionality and usability, require modification and refinement for a future full-scale study.

### Key findings and design implications for a full-scale study

#### Identification and recruitment of participants

By using primary care general practice registers, we were able to identify sufficient potentially eligible participants; however, the proportion of non-responders to invitation was high (331/442 (70%)), for reasons unknown. The proportion of mailed participants enrolled into the study was 14/442 (3.2%). This recruitment rate is notably less than the 12% response from our recent pen-and-paper diary study (unpublished data) and appears relatively inefficient. Previous studies have demonstrated that concurrent pen-paper or Internet questionnaire completion options do not result in better response rates [28, 29]; however, the loss of eligible potential participants due to the requirement to transition from offline to online is one plausible contributing factor for our low response. By approaching people via post, the extent to which people may have chosen not to express interest in this study due to lack of Internet access or literacy cannot be known. Supplementing this approach with offline community advertising and/or online social media advertising, directing people to a self-enrolment page on the study website, may help to target individuals who are more inclined or willing to take part in online research [30, 31]. In terms of generating the sample, these parallel processes could facilitate recruitment of individuals from across wider geographical locations. Over-zealous exclusion criteria contributed to lower recruitment. Close inspection of our eligibility criteria indicated that we subsequently deemed 76 potential participants ineligible for a combination of the following reasons: current hot swollen knees, knee injury in the last 3 months bad enough to see a doctor, knee replacement or on waiting list, corticosteroid injection into the knee in the last 3 months, and knee surgery in the last 3 months. Had we relaxed these exclusion criteria and accepted into the study all people who (i) met the general practice search and screen inclusion criteria and (ii) had daily access to email and the Internet and could complete questionnaires in English, this would have yielded a revised eligibility rate of 66.4% (95% CI 56.8, 74.7). Expecting that approximately half of these individuals would provide e-Consent and complete a baseline questionnaire, the revised estimate for overall recruitment rate would be 7.9% (95% CI 5.8, 10.8).

People with hot swollen knees were excluded on the basis that these characteristics may represent more acute red flag presentations such as sepsis or other arthritis conditions. Assuming that such occurrences will be rare within a community-based sample, this criterion could have been relaxed. Furthermore, people with OA who have warm knees who experience swelling may be individuals more likely to experience flare-ups [32]. Although our other eligibility criteria improve the homogeneity of the sample with regard to knee characteristics, an alternative strategy for a future full-scale study would invite all of the 69 interested potential participants and subsequently evaluate the impact of these criteria with sensitivity analyses.

The technical process of obtaining e-Consent was adequate, and there were no reported problems with functionality. Five participants opted not to provide consent, and a further eight did not respond to the welcome email for reasons unknown. To encourage e-Consent completion and enrolment among those who initially express interest, a reminder email could be built into a future full-scale study. A small number of participants experienced difficulties accessing the website during early stages of the study, and their issues were handled in real-time. These issues contributed to the decision made by two consenting participants to withdraw from the study.

#### Retention, follow-up, and flare-up notification

Based on completing one scheduled follow-up questionnaire, retention was 57%. However, the overall symptom severity of the sample was mild and had we included people experiencing hot swollen knees, their increased symptoms may have led to more flare-up notifications and general engagement with the study.

The function designed to capture and redirect participants experiencing a flare-up at a scheduled follow-up proved effective. For the flare-up notification process more broadly, our procedures may have impeded some participants’ ability to report flare-ups. Four participants reported a flare on the day of onset. They then received an automated response inviting them to return the next day to ensure their flare-up lasted for 24 h. None of these participants returned the next day. It is impossible to know whether this is due to resolution of symptoms or failure to return to the website. The decision to invite participants to complete an event-driven questionnaire if flare-up onset was within 3 days was designed to aid recall of the last 7 days from onset. It may have been more appropriate to allow all participants to complete an event-driven questionnaire on the day a flare-up is reported, then apply a case definition, such as sustained for at least 24 h, by using the daily flare questionnaire data during analysis. This more pragmatic approach would be acceptable given that there is currently no agreed knee OA flare-up definition within the OA community [6]. It would appear that more participants wished to notify us of a flare-up than were recorded during the study period, although it cannot be ruled out that some participants may have logged a flare in error. For example, by clicking on the flare notification icon whilst exploring the website. An ‘are you sure?’ function could be added at this point to reduce the potential for people to report flare-ups inadvertently. Whilst the overall recorded proportion of flare-related contacts was encouraging at 50%, nearly half of the flare-ups were reported at scheduled follow-up time points; hence, the capture of flare data may be enhanced with a more effective reminder process, for example by sending bi-weekly text message reminders for the study duration.

#### Data quality and methodological sub-studies

The decision to include a daily measurement sub-study was designed to enable sensitivity analysis relating to the potential for recall bias between the scheduled and event-driven questionnaire comparisons. By capturing additional daily prospective pain scores, these data could be compared to the retrospectively reported data within the main analysis to quantify the amount of potential bias that could be attributed to recall. Given the poor retention over the study period, it is possible that this extra burden of engagement could have adversely affected follow-up. If this process was removed from a full-scale study, recall and recall bias could be handled with other less-burdensome techniques. Firstly, by reducing the recall period from the last 7 days to the last 3 days: This would help with the general cognitive effort required to accurately recall issues over the last few days and may also improve response at scheduled follow-up time points. Secondly, by adding ‘normal’ frequency of exposure to the baseline questionnaire for key potential triggers being investigated, an additional control sample could be generated to compare against the case period. This would mean that multiple control sampling strategies are incorporated into the design. These could include within-person case-crossover comparisons between the day before the flare-up started in the flare-up questionnaire (case-period) and three control-period samples: (i) the 2 days prior in the event-driven questionnaire, (ii) 1 or more 3-day periods within the scheduled questionnaire(s), or (iii) the normal frequency of exposure in the baseline questionnaire [33]. These could be compared and contrasted with sensitivity analyses to examine the potential influence of recall bias, and the optimal control period selected as the one that maximises the exposure odds ratio [34]. This approach could also negate the need to evaluate recall bias using objective weather data, which could not be evaluated in our study. Poor retention and engagement over a 9-week period also indicates that high-quality case-control comparisons may be more efficiently obtained by increasing sample size over a short period of follow-up, rather than a more extended period. A further advantage of the case-crossover design is that the data can be analysed as a cohort study when ‘normal’ frequency of exposure at baseline is collected.

Although the number of people responding to the post-study questionnaire was small (*n* = 6), more people expressed willingness to receive an MRI scan during a flare-up (*n* = 4), rather than a joint aspiration (*n* = 2). This observation may reflect the more invasive nature of joint aspiration.

### Strengths and limitations

Major strengths of this study were patient involvement and the use of clinician and patient meetings for interpreting the findings and evaluating whether and how to transition to full-scale study. A limitation was the website’s incompatibility with smartphone use. This may have restricted the flexibility of questionnaire completion. A future full-scale study could benefit from smartphone compatibility, which may improve follow-up retention, particularly for the brief daily questionnaires during flare-up periods. Six participants reported a flare-up at baseline. This may have affected the way their general health-related questions were completed. Conducted on a larger scale, the impact of this on any derived estimates would need to be quantified with sensitivity analyses. Finally, our understanding of why people chose not to participate may have been enhanced further had we included a nested qualitative study.

## Conclusions

Feasibility and pilot studies are most typically undertaken in the context of developing full-scale intervention trials [35], but they can be valuable for observational studies too, particularly where there are important uncertainties in their design and implementation. In this study, our recruitment rate of 3% is substantially lower than comparable rates for offline questionnaire-based studies. Proposed solutions to this were suggested by an evaluation of process in the current study and from previous relevant studies. However, the outcome of implementing these in future full-scale study is necessarily uncertain.

## Abbreviations

CI: Confidence intervals
COPD: Chronic Obstructive Pulmonary Disease
GMT: Greenwich Mean Time
GPPAQ: General Practice Physical Activity Questionnaire
GPSS: General practice search and screen
IQR: Interquartile range
KOOS-PS/(Qol): Knee injury and Osteoarthritis Outcome Score-Physical Function Short Form (Quality of Life)
MRI: Magnetic resonance imaging
NRS: Numerical rating scale
OA: Osteoarthritis
PAG: Patient advisory group
PCRF: Patient-completed reply form
PIS: Participant information sheet
